# Traditional Cantonese diet and nasopharyngeal carcinoma risk: a large-scale case-control study in Guangdong, China

**DOI:** 10.1186/1471-2407-10-446

**Published:** 2010-08-20

**Authors:** Wei-Hua Jia, Xiang-Yu Luo, Bing-Jian Feng, Hong-Lian Ruan, Jin-Xin Bei, Wen-Sheng Liu, Hai-De Qin, Qi-Sheng Feng, Li-Zhen Chen, Shugart Yin Yao, Yi-Xin Zeng

**Affiliations:** 1 State Key Laboratory of Oncology in Southern China, Guangzhou, 510060, China; 2 Department of Experimental Research, Sun Yat-Sen University Cancer Center, Guangzhou, China; 3 School of Public Health, Sun Yat-Sen University, Guangzhou, 510080, China; 4 Department of Dermatology, University of Utah School of Medicine, Salt Lake City, UT 84132, USA; 5 Department of Epidemiology, Johns Hopkins Bloomberg School of Public Health, Baltimore, MD 21205, USA

## Abstract

**Background:**

Nasopharyngeal carcinoma (NPC) is rare in most parts of the world but is a common malignancy in southern China, especially in Guangdong. Dietary habit is regarded as an important modifier of NPC risk in several endemic areas and may partially explain the geographic distribution of NPC incidence. In China, rapid economic development during the past few decades has changed the predominant lifestyle and dietary habits of the Chinese considerably, requiring a reassessment of diet and its potential influence on NPC risk in this NPC-endemic area.

**Methods:**

To evaluate the association between dietary factors and NPC risk in Guangdong, China, a large-scale, hospital-based case-control study was conducted. 1387 eligible cases and 1459 frequency matched controls were recruited. Odds ratios (ORs) and the corresponding 95% confidence intervals (CIs) were estimated using a logistic regression model, adjusting for age, sex, education, dialect, and habitation household type.

**Results:**

Observations made include the following: 1) consumption of canton-style salted fish, preserved vegetables and preserved/cured meat were significantly associated with increased risk of NPC, with enhanced odds ratios (OR) of 2.45 (95% CI: 2.03-2.94), 3.17(95% CI: 2.68-3.77) and 2.09 (95% CI: 1.22-3.60) respectively in the highest intake frequency stratum during childhood; 2) consumption of fresh fruit was associated with reduced risk with a dose-dependent relationship (p = 0.001); and 3) consumption of Canton-style herbal tea and herbal slow-cooked soup was associated with decreased risk, with ORs of 0.84 (95% CI: 0.68-1.03) and 0.58 (95% CI: 0.47-0.72) respectively in the highest intake frequency stratum. In multivariate analyses, these associations remained significant.

**Conclusions:**

It can be inferred that previously established dietary risk factors in the Cantonese population are still stable and have contributed to the incidence of NPC.

## Background

Although rare in most parts of the world, nasopharyngeal carcinoma (NPC) is a common malignancy in southern China, especially in the Guangdong province [1,2]. The incidence rate for males is more than 20 per 100,000 person-years and is as high as 25 to 40 per 100,000 person-years in some areas bordering the Xijiang River and the Pearl River [1-4]. Within China, the incidence of NPC varies up to 50-fold between regions, with rates generally increasing from northern China (e.g., Beijing and Tianjin) to southern China; the rates in Chinese men in the most northern provinces are no higher than 3 per 100,000 person-years [1,2]. Reports indicate that southern Chinese who migrate to intermediate-risk areas, such as Southeast Asia, or to low-risk areas, such as the United Kingdom, Australia and the United States, continue to have a high incidence of NPC [1,5-10]. However, those who reside for a longer period of time in low-risk areas and their succeeding generations born in the West, have a decreased risk for NPC [8-10]. These results suggest that environmental factors inherent in the traditional southern Chinese culture are responsible for the unusually high incidence of NPC in southern China.

In the early 1970 s, Ho suggested that ingestion of salted fish, a traditional southern Chinese food favoured by the Cantonese, might be a cause of the high incidence of NPC in the region [11]. Subsequent studies have consistently reported that consumption of salt-preserved fish in NPC-endemic areas of Guangdong and Guangxi in Southern China [12-14] is strongly associated with risk of NPC. Consistent results were also observed in Tianjin [15] and Shanghai [16], the areas with low and moderate NPC risk in North and East China respectively. In addition to salted fish, exposure to other preserved food products, such as salted shrimp paste, fermented soybean paste and various types of salted vegetables, is related to NPC risk in Chinese[13,15]. In a current study in Guangxi, a second risk area of Southern China, salted fish consumption was still observed to be related to NPC risk [14].

Herbal medicine is part of traditional Chinese medicine. It has been widely used in formal clinical practice in China for many centuries. Although several case-control studies reported a two- to four-fold excess risk of NPC in association with the use of traditional herbal medicines in some Asian populations, no such association has been reported in southern China. When specific herbal plants were examined, no single plant species appeared to be responsible for the observed association between herbal medicine and NPC. Notably, herbal drugs have also been commonly consumed in the Cantonese diet as a component of Cantonese-style slow cooked soup and tea. However, until now herbal medicines as dietary component have not been evaluated in the context of their potential association with NPC risk.

In China, rapid economic development during the past two to three decades has considerably changed the lifestyle and dietary habits of the Chinese population. These changes have encouraged us to reassess dietary habits and their potential influence on NPC risk in this NPC-endemic area. In Guangdong, a province showing the highest incidence rate in China and different lifestyles from adjacent areas, the latest report on dietary factors and their relationship with NPC risk was conducted in 1989[13]. With the new biological technology development, it is important and necessary to organize a new epidemiological investigation, in which, we not only focused on lifestyle and other environmental factors, but also collected whole blood samples for each individual so as to develop a systematic genetic study simultaneously. Base on the project, our new findings of NPC susceptibility genes using GWAS approach have been reported [17]. Gene-environment-EBV interaction on the aetiology of NPC will be investigated in depth in the near future.

This study was engaged in re-evaluating previously reported dietary risk factors and in trying to find new potential factors in Guangdong. We observed for the first time that Cantonese herbal tea and herbal slow-cooked soup decrease NPC risk.

## Methods

### Subjects

This is a hospital-based case-control study. Cases were identified from the medical records of the Sun Yat-Sen University Cancer Center, the largest centre for cancer prevention and treatment in southern China. The centre is located in Guangzhou, the capital of Guangdong province, and it draws patients from all areas of Guangdong Province. The Sun Yat-Sen University ethical committee approved the study protocol. Eligibility criteria included the following: 1) histological confirmation of NPC, 2) age less than 80 years, 3) no previous diagnosis of or treatment for NPC, and 4) residence in Guangdong province. Among all eligible NPC patients identified between October 2005 and October 2007. 1387 eligible cases were recruited in the study. A total of 61 patients could not answer the questions qualitatively because of severe side effects associated with radiotherapy. Therefore, 95.8% were enrolled in the study.

Controls were frequency-matched to cases by age, sex, education, dialect, household type (rural or urban). The controls were recruited from people who requested health examinations in the centers of physical examination of the largest general hospitals in Guangdong province. All subjects were required to be Guangdong residents without any history of malignancy. Qualified participants were also required to have the physical and mental ability to complete the interview. A total of 1,459 qualified controls were included in the study.

For each participant, we provided services including serologic testing for anti-VCA-IgA antibody, otorhinolaryngologic examinations, and medical consultations performed by physicians from SYSUCC. Those who were positive for anti-VCA-IgA antibody were referred to the NPC department at SYSUCC, where they underwent fiberoptic endoscopy performed by an otorhinolaryngologist.

### Data collection

Interviews were conducted face-to-face by trained interviewers employing a structured questionnaire in hospitals for case patients and in physical examination centres for control subjects. Informed consent was obtained from each individual before the interview began. Data were collected for demographic characteristics (age, gender, education, dialect and household type etc.), lifestyle information and family history of NPC among first degree relatives. Lifestyle information included dietary habits during childhood (defined as prior to twelve years of age) and adulthood. The dietary habits involving Chinese herbal plants included the consumption of Canton-style herbal tea and herbal slow-cooked soup, both of which involve about 30 herbal plants. Regarding food, intake frequency of salted fish, salted vegetables, preserved/cured meat, fermented pastes and fresh fruits were collected; subjects were asked to choose from five intake frequency categories: never, sometimes, monthly, weekly and daily.

### Data analysis

Statistical analyses were conducted using STATA software, version 10.0. In the analyses, successive intake frequencies were pooled together to eliminate rare categories. Odds ratios (ORs) and corresponding 95% confidence intervals (CIs) were estimated using a logistic regression model, adjusting for age, sex, education, dialect and habitation household type. Linear trend tests for exposure-disease associations were performed for continuous variables.

## Results

The demographic characteristics and socioeconomic status of the study population are shown in Table 1. A total of 1,387 eligible cases and 1,459 healthy controls were recruited and used in the analyses. As expected due to our matching strategy, the distribution by age, sex, education, dialect and rural/urban resident type were comparable between cases and controls. All of the subjects were of Han ethnicity.

**Table 1:** Demographic characteristics and socioeconomic status of the study populations

Variables	Case (%)	Control (%)	*P*-value
Sex			
Male	1025(74)	1038(71)	0.100
Female	362(26)	421(29)	
Age			
Mean	46.92	47.34	
(SD)	11.34	11.64	
< 30	84(6)	78(5)	0.564
30-40	323(23)	352(24)	
41-50	442(32)	431(30)	
51-60	371(27)	414(28)	
> 60	167(12)	184(13)	
Dialect			
Hakka	219(16)	206(14)	0.603
Cantonese	917(66)	971(67)	
Hokkien	133(10)	146(10)	
Others	116(8)	132(9)	
Education			
None or primary school	282(20)	297(20)	0.998
Secondary school	373(27)	391(27)	
High school	459(33)	479(33)	
University or more	269(20)	286(20)	
Household type			
Rural	965(70)	1018(70)	0.874
Urban	412(30)	429(30)	

Table 2 presents the relationship between dietary factors and NPC risk in the case-control dataset. Consumption of salted fish, salted vegetables and preserved/cured meat were all risk factors for NPC, with elevated ORs of 2.45 (95% CI: 2.03-2.94), 3.17(95% CI: 2.68-3.77) and 2.09 (95% CI: 1.22-3.60) respectively, for the most frequently consumption stratum during childhood. Consumption of fresh fruit was inversely associated with NPC risk, with ORs of 0.38 (95% CI: 0.28-0.50) and 0.12 (95% CI: 0.09-0.16) for monthly and "weekly or more" stratum during childhood respectively. While all these associations were observed both in adulthood and childhood, the effects of consumption of salted fish and fresh fruit were stronger in childhood than in adulthood. However, preserved/cured meat has a stronger effect in adulthood than in childhood. When samples were stratified by sex, region or age group, the analyses did not yield significant evidence of heterogeneity in the odds ratios (data not shown).

**Table 2:** Association of dietary factors with NPC

Intake frequency *	Adulthood		Childhood	
		
	Case/Ctrl	OR(95%CI)^#^	Case/Ctrl	OR (95%CI)^#^
Salted fish				
Less than monthly (ref)	1085/1272	1.0	724/1075	1.0
Monthly	162/80	2.39(1.80-3.17)	236/116	3.04(2.38-3.87)
Weekly or more	128/98	1.58(1.20-2.09)	415/254	2.45(2.03-2.94)
*P *_trend_		< 0.001		< 0.001
Salted vegetables				
Less than monthly (ref)	970/1252	1.0	551/962	1.0
Monthly	218/85	3.31(2.54-4.31)	204/129	2.72(2.13-3.48)
Weekly or more	190/113	2.20(1.71-2.82)	624/355	3.17(2.68-3.77)
*P *_trend_		< 0.001		< 0.001
Preserved/cured meat				
Less than monthly (ref)	606/1256	1.0	859/1242	1.0
Monthly	170/46	7.80(5.33-11.39)	55/49	1.67(1.09-2.54)
Weekly or more	157/37	9.69(6.39-14.70)	35/27	2.09(1.22-3.60)
*P *_trend_		< 0.001		0.001
Fermented pastes				
Less than monthly (ref)	1243/1301	1.0	1056/1203	1.0
Monthly	76/44	1.82(1.24-2.66)	149/80	2.12(1.59-2.82)
Weekly or more	56/106	0.57(0.41-0.79)	174/164	1.22(0.97-1.54)
*P *_trend_		0.157		0.001
Fresh fruits				
Less than monthly (ref)	299/234	1.0	1176/797	1.0
Monthly	138/91	1.22(0.85-1.76)	92/166	0.38(0.28-0.50)
Weekly or more	936/1126	0.63(0.51-0.77)	106/481	0.12(0.09-0.16)
*P *_trend_		0.157		0.001

* Intake frequency was categorised as follows: less than monthly, < once per month; monthly, once per month to once per week; weekly or more, ≥ once per week.

^# ^OR, odds ratio calculated among matched case and control groups, adjusting for sex, age, education, dialect and household type.

Interestingly, a reduced risk of NPC was newly observed for the consumptions of Canton-style herbal tea and herbal slow-cooked soup (Table 3). For herbal slow-cooked soup, both intake frequency and duration were associated with reduced risk with a dose-dependent relationship. ORs were 0.49 (95% CI: 0.27-0.87) and 0.58 (95% CI: 0.47-0.72) for "monthly" and "weekly or more" group compared with reference of "less than monthly" group, P _trend _< 0.0001. ORs were 0.67 (95% CI: 0.50-0.90), 0.53(95% CI: 0.42-0.67) and 0.22 (95% CI: 0.18-0.28) respectively for consumption duration of year 1-9, 10-19, ≥20 compared with reference of "less than monthly" group, P _trend _< 0.0001.

**Table 3:** Relationship between dietary habits with Chinese herbs and NPC

Intake frequency * and duration	Case/Ctrl	OR (95%CI)^#^	***P ***_**trend**_
Herbal tea habit			
Less than monthly (ref)	439/347	1.0	
Monthly	425/717	0.46(0.38-0.56)	< 0.0001
Weekly or more	369/347	0.84(0.68-1.03)	
Herbal tea (years)			
Less than monthly (ref)	439/347	1.0	
1-9	237/237	0.81(0.64-1.03)	< 0.0001
10-19	88/320	0.22(0.16-0.29)	
≥20	146/457	0.25(0.19-0.31)	
Slow-cooked soup habit			
Less than monthly (ref)	313/214	1.0	
Monthly	39/42	0.49(0.27-0.87)	< 0.001
Weekly or more	996/1184	0.58(0.47-0.72)	
Slow-cooked soup (years)			
Less than monthly (ref)	313/214	1.0	
1-9	146/142	0.67(0.50-0.90)	< 0.0001
10-19	310/380	0.53(0.42-0.67)	
≥20	250/659	0.22(0.18-0.28)	

* Intake frequency was categorised as follows: less than monthly, < once per month; monthly, once per month to once per week; weekly or more, ≥ once per week; daily, ≥ once per day.

^# ^OR, odds ratio calculated among matched case and control groups, adjusting for sex, age, education, dialect and household type.

In a multivariate logistic regression analysis (Table 4), salted fish, salted vegetables during childhood and salted vegetables, preserved/cured meat during adulthood increased the risk of NPC, while fresh fruit consumption during childhood decreased the risk. Moreover, herbal tea and slow-cooked soup remained significantly associated with decreased risk of NPC in the multivariate logistic regression model (Table 4).

**Table 4:** Multivariate analyses of dietary factors*

Dietary Factors	OR^#^	95%CI	*P*-value
Salted fish in childhood			
Less than monthly(ref)	1.00		
Monthly	2.42	1.65-3.54	< 0.001
Weekly or more	1.57	1.16-2.13	0.003
Salted vegetables in childhood			
Less than monthly(ref)	1.00		
Monthly	1.28	0.87-1.89	0.215
Weekly or more	1.81	1.37-2.40	< 0.001
Salted vegetables in adulthood			
Less than monthly(ref)	1.00		
Monthly	2.23	1.46-3.40	< 0.001
Weekly or more	1.79	1.19-2.68	0.005
Fresh fruits in childhood			
Less than monthly(ref)	1.00		
Monthly	0.31	0.20-0.47	< 0.001
Weekly or more	0.13	0.09-0.18	< 0.001
Preserved and cured meat in adulthood			
Less than monthly(ref)	1.00		
Monthly	8.32	5.46-12.69	< 0.001
Weekly or more	12.39	7.60-20.19	< 0.001
Herbal tea habit			
Less than monthly(ref)	1.00		
Monthly	0.35	0.27-0.47	< 0.001
Weekly or more	0.57	0.41-0.78	0.001
Slow-cooked soup habit			
Less than monthly(ref)	1.00		
Monthly	0.44	0.21-0.93	0.031
Weekly or more	0.49	0.37-0.65	< 0.001

*Logistic regression model was built by a stepwise forward method with inclusion criteria p ≤ 0.01.

Both of the dietary consumptions in childhood and adulthood were used in the multivariate analysis.

^#^OR, odds ratio calculated among matched case and control groups, adjusting for sex, age, education, dialect and household type.

## Discussion

Results from the present study indicate that consumption of salted fish, salted vegetables and preserved/cured meat is a risk factor for NPC, while consumption of fresh fruit is inversely associated with NPC risk. Consumption of salted fish has been validated as a significant risk factor for NPC in many studies [13-15,18,19]. Several nitrosamines have been detected in samples of Chinese salted fish, most of which are capable of inducing nasal cavity tumours in experimental animals [20-22]. In studies of Chinese populations, the relative risk of NPC associated with weekly consumption of salted fish ranged from 1.4 to 3.2 when compared with no or rare consumption; for daily consumption, the relative risk ranged from 1.8 to 7.5 [23]. We have not found any apparent difference in the magnitude of the association between the present study and the previous studies. In addition, previous studies observed that childhood exposure seems more strongly related to NPC risk than adulthood exposure [12,13,15,18,24-26]. We obtained a consistent result in this study, whereby OR was 2.45 (2.03-2.94) in childhood vs. 1.58 (1.20-2.09) in adulthood at consumption frequency of weekly or more.

Besides salted fish, exposure to other preserved foods, such as salted vegetables and preserved meat, was also found to be associated with an increased risk of NPC. This is fairly consistent with previous publications [13,15,16,18,23]. It has been reported that mouldy bean curd, salted shrimp paste, salted eggs and various preserved vegetables were associated with an increased risk of NPC. Intake of preserved foods has also been shown to be associated with NPC risk among Arabs in Maghrebian countries [27,28]. Similar to Chinese salted fish, these NPC-associated preserved foods contain carcinogenic nitrosamines and other genotoxic substances [29]. Noteworthy, we have not found convinced evidence that fermented pastes (bean, fish or shrimp paste) increase the risk of NPC.

Our data demonstrate that NPC patients had ingested significantly fewer fresh fruits than control subjects, which was consistent with previous several reports, especially for childhood exposures [12,13,15,16,18,25].

Fresh fruit contains high levels of vitamin C, which not only blocks nitrosamine formation *in vivo *but also inhibits mutagenesis and carcinogenesis *in vitro *and inhibits tumour cell growth and carcinogen-induced DNA damage [30]. These findings offer a biological rationale for the observed protective effect of fresh fruit in NPC development as reported by previous studies [31].

Interestingly, intake of herbal tea and herbal slow-cooked soups are found to be associated with decreased risk of NPC. Aboriginal habitants of the Guangdong frequently consume Canton-style herbal tea, guided by the belief that it can heal or prevent common ailments like the flu and sore throats. The tea contains traditional Chinese medicinal herbs such as *mulberry (Morus alba) leaf, yellow or white chrysanthemum (Chrysanthemum) flowers, prunella (Prunella vulgaris) spike, roughhaired holly (Radix Ilicis Asprellae) root, ural licorice (Radix Glycyrrhizae) root *and others. The type of herbs varies among several brands of herbal teas or homemade herbal teas depending on their medicinal purposes.

Unlike herbal tea, herbal slow-cooked soup contains meat and is normally simmered for several hours. The herbs in a typical slow-cooked soup include *Coix Seed (Semen Coicis), Lily Bulb (Lilium Lancifolium), Gordon Euryale Seed (Semen Euryales), Dioscoreae Rhizome (Rhizoma Dioscoreae Oppositae), Fragrant Solomonseal Rhizome (Rhizoma Polygonati Odorati), lotus seed (Semen Nelumbinis), Chinese Date (Fructus Jujubae) *and others.

Studies conducted on Filipino populations contradict our findings; these studies suggest that herbal medicines are independently associated with an increased risk of NPC[32] and that herbal medicines interact with EBV through a direct proliferative effect on EBV-transformed cells[33]. One study conducted in Taiwan also observed an association between herbal medicines and increased risk of NPC [34]. However, studies conducted by Yu et al. in mainland China failed to detect an independent effect of herbal medicine use on NPC risk [13,35]. Specific herbal medicines were examined, but no single plant family appeared to be responsible for the association observed between herbal medicine use and NPC. The possible explanation of the disparity in the study results may be that the term "herbal medicine" is too broad; the involved herbal plants could be heterogeneous between studies and/or within a study. On the other hand, the herbal tea and herbal slow-cooked soup investigated in the present study are Canton-style dietary staples that involve a limited number of herbal plants without remarkable variance throughout the Guangdong province. Thus it is a more homogeneous variable than traditional herbal medicines treated as a whole. Several studies have shown that some Chinese herbal plants can play their anticancer role by inducing apoptosis and differentiation [36,37], by enhancing the immune system, inhibiting angiogenesis or reversing multi-drug resistance (MDR)[34]. It is noteworthy that Prunella vulgaris extracts were shown to have anti-viral, anti-proliferative and immunostimulatory effects [38,39]. Coix seed has inhibitory effects on Epstein-Barr virus activation and has anti-tumour promoting activities.

It is possible that the herbal tea and herbal slow-cooked soups consumed in Guangdong province contain herbal plants that have similar anticancer capabilities. However, it is also possible that this association came from the fact that part of our controls were recruited from individuals taking health examinations, who are more concerned about their health and hence tend to consume more herbal tea or soup. Therefore, the relationship between dietary habits including Chinese medicinal herbs and NPC should be interpreted with caution, and further in-depth analyses and experiments are required.

We have several limitations in this study. Similar to case-control approach, recall bias is inevitable. The data on intake frequency inevitably include a certain percentage of misclassification, particularly among older subjects when recalling the past. For example, when asked about past intake frequency, some people could not remember exactly whether it was monthly or weekly. Perhaps this inexact recall partly explains the observation that the highest intake frequencies of salted fish and fermented paste (bean, fish or shrimp paste) were associated with lower odds ratios. In addition, we did not obtain the precise frequencies of dietary consumptions, for example, how many times per day, and none evaluation for their contribution to NPC risk were provided. Moreover, the study population may be aware of some of the known NPC risk factors such as salted fish. Thus, this factor may be another source of recall bias.

Socioeconomic status (SES) could affect our subject recruitment in several ways. Recruitment of controls from people taking health examinations may also yield ascertainment bias towards higher SES in the controls. However, SES was carefully controlled not only in frequency matched design of cases and controls' recruitment, but also in data analysis. With these steps, the confounding effect of SES was minimised to a great extent. However, the possibility that residual SES confounding may exist cannot be excluded.

## Conclusions

Taken together, this study has provided a clearer view of the dietary risk factors for NPC. Similar to previous studies, consumption of salted fish and other preserved foods increased the risk of NPC, while consumption of fresh fruit reduced the risk. Further, we found that cases consumed less herbal tea and herbal slow-cooked soup than controls. Further study is needed to integrate molecular pathology with epidemiologic study for the identification of the carcinogens.

## Competing interests

The authors declare that they have no competing interests.

## Authors' contributions

WHJ is the guarantor of the study. She designed the study and was the main author of the manuscript. XYL performed data analysis and drafted the manuscript. BJF and HLR participated in data analysis and manuscript revising, JXB participated in data cleaning and analysis, WSL verified and cleaned the data, HDQ verified the data and revised the manuscript. QSF and LZC conducted sample collection and the interviews, SYY and YXZ participated in the design of the study. All authors read and approved the final manuscript.

## Pre-publication history

The pre-publication history for this paper can be accessed here:

http://www.biomedcentral.com/1471-2407/10/446/prepub
